# The Epigenetic Controller Lysine-Specific Demethylase 1 (LSD1) Regulates the Outcome of Hepatitis C Viral Infection

**DOI:** 10.3390/cells12212568

**Published:** 2023-11-03

**Authors:** Georgia Papadopoulou, Stavroula Petroulia, Eirini Karamichali, Alexios Dimitriadis, Dimitrios Marousis, Elisavet Ioannidou, Panagiota Papazafiri, John Koskinas, Pelagia Foka, Urania Georgopoulou

**Affiliations:** 1 Molecular Virology Laboratory, Hellenic Pasteur Institute, 11521 Athens, Greece; 2 Division of Animal and Human Physiology, Department of Biology, National and Kapodistrian University of Athens, 15784 Athens, Greece; 3 Molecular Biology and Immunobiotechnology Laboratory, Hellenic Pasteur Institute, 11521 Athens, Greece; 4 2nd Department of Internal Medicine, Medical School of Athens, Hippokration General Hospital, 11521 Athens, Greece

**Keywords:** KDM1A, lysine-specific demethylase, hepatitis C virus, interferon, IFITM3, epigenetics, endocytosis, entry, lysosome

## Abstract

Hepatitis C virus (HCV) alters gene expression epigenetically to rearrange the cellular microenvironment in a beneficial way for its life cycle. The host epigenetic changes induced by HCV lead to metabolic dysfunction and malignant transformation. Lysine-specific demethylase 1 (LSD1) is an epigenetic controller of critical cellular functions that are essential for HCV propagation. We investigated the putative role of LSD1 in the establishment of HCV infection using genetic engineering and pharmacological inhibition to alter endogenous LSD1 levels. We demonstrated for the first time that HCV replication was inhibited in LSD1-overexpressing cells, while specific HCV proteins differentially fine-tuned endogenous LSD1 expression levels. Electroporation of the full-length HCV genome and subgenomic replicons in LSD1 overexpression enhanced translation and partially restored HCV replication, suggesting that HCV might be inhibited by LSD1 during the early steps of infection. Conversely, the inhibition of LSD1, followed by HCV infection in vitro, increased viral replication. LSD1 was shown to participate in an intriguing antiviral mechanism, where it activates endolysosomal interferon-induced transmembrane protein 3 (IFITM3) via demethylation, leading endocytosed HCV virions to degradation. Our study proposes that HCV-mediated LSD1 oscillations over countless viral life cycles throughout chronic HCV infection may promote epigenetic changes related to HCV-induced hepatocarcinogenesis.

## 1. Introduction

Lysine-specific demethylase 1 (LSD1/KDM1A), a member of the amine oxidase family, catalyses the enzymatic demethylation of mono- and dimethylated lysine residues [1]. LSD1 is well known for being involved in the epigenetic regulation of gene expression by targeting Lys4 and Lys9 sites on histone H3 (H3K4me1/2 and H3K9me1/2), acting either as a repressor or as an activator of gene transcription, depending on which cofactors are available in the cellular microenvironment at any given time [2]. LSD1 also targets non-histone proteins. The tumour suppressor gene p53 was the first to be identified as a substrate of LSD1 activity [3]. Other transcription factors, such as DNA methyltransferase 1 (DNMT1), the signal transducer and activator of transcription 3 (STAT3), and E2F transcription factor 1 (E2F1), have since been found to be LSD1 substrates [4].

Additionally, LSD1 acts as a key regulator in a plethora of physiological cell functions, with embryonic development, haematopoiesis, circadian cycle, DNA damage response, mitochondrial function and lipid metabolism being only a few examples [5]. Notably, many other cellular processes, including cell proliferation and differentiation, regulation of the stem cell state, epithelial–mesenchymal transition (EMT) and malignant transformation, are closely related to elevated levels of LSD1, which have been observed in a variety of malignancies [6].

Among several types of cancer, LSD1 expression was found significantly increased in hepatocellular carcinoma (HCC) compared with adjacent non-neoplastic liver tissue, thereby rendering LSD1 a diagnostic marker and therapy target. On top of that, its elevated expression in higher-tumour-stage and -grade tissues suggested that it may be associated with disease progression and poor survival [7]. Although alcohol- and non-alcoholic steatohepatitis (NASH)-related HCC is growing in numbers nowadays, chronic viral hepatitis remains a major cause of liver cancers worldwide [8].

Interestingly, it has been suggested that LSD1 is involved in the life cycle of numerous DNA viruses, contributing to the establishment of infection. For example, it plays an important role in Herpes Simplex Virus-1 (HSV-1) productive infection and virus reactivation from latency [9], and takes part in the activation of the Hepatitis B Virus (HBV) cccDNA chromatin state [10]. However, the antiviral properties of LSD1 against RNA viruses have only recently been described. In particular, LSD1 overexpression impairs Vesicular Stomatitis Virus (VSV) replication by promoting retinoic acid-inducible gene I (RIG-I) poly-ubiquitination [11] and restricts Influenza A Virus (IAV) infection after interferon stimulation [12]. The above-mentioned studies highlight the diverse role of LSD1 in viral propagation and infection, which largely depends on the particular viral life cycle.

Hepatitis C virus (HCV) is a small RNA virus which manages to complete a unique life cycle with well-defined steps that have been studied thoroughly, by using just ten multifunctional proteins [13]. Without being lytic, HCV causes devastating human disease. HCV infection is among the major causes of chronic hepatitis, cirrhosis and HCC. HCV causes persistent infection in the majority of cases, which goes undetected for many years [14]. Around 1.5 million new cases of HCV are reported each year, with an estimated 58 million people carrying the infection worldwide. Hepatitis C was responsible for approximately 290.000 deaths in 2019, primarily from cirrhosis and HCC [15]. Although direct-acting antivirals (DAAs) have been shown to eradicate the virus and to increase sustained virological responses (SVRs) in 95% of patients [16], the data on DAA-induced cure and de novo HCC development or recurrence remain contentious, especially in cases of advanced fibrosis and cirrhosis [17,18].

Despite the fact that HCV does not cause gene mutations via insertion into host chromosomes, it strongly drives hepatocarcinogenesis, mostly through the action of key viral proteins, such as HCV core and NS5A, the induction of oxidative stress and chronic deregulation of the immune system, host metabolism and cell signalling [19,20]. Furthermore, it has been shown that HCV infection induces epigenetic alterations that strongly influence host-cell gene transcription and methylation state, as well as other molecular pathways involved in the completion of the viral life cycle, the establishment of successful infection and chronicity. A link between epigenetic regulation and cancer development has already been suggested for other oncogenic viruses, such as Human Papilloma Virus (HPV) and cervical carcinogenesis [21] or HBV and HCC [22]. Thus, it is possible that epigenetically driven changes in hepatic gene expression throughout chronic HCV infection could predispose susceptible individuals to HCC occurrence. Importantly, even after HCV eradication with DAA treatment, these modifications may persist as an epigenetic signature, thus affecting molecular pathways involved in HCC development and progression [23].

The deregulation of LSD1 expression levels in virally induced HCC hints towards a possible role of LSD1 in the establishment of HCV infection, which is the first step towards HCC development. Our study aimed to investigate the existence of such a mechanism that could render LSD1 a key player for the completion of the HCV life cycle.

## 2. Materials and Methods

### 2.1. Materials

Human hepatoma Huh7.5 and Huh7-Lunet cells were supplied by Dr. Ralf Bartenschlager (University of Heidelberg, Heidelberg, Germany). Interferon beta-1a was from Merck and cell culture media were from Gibco (Thermo Fisher Scientific, Waltham, MA, USA). A Pierce™ Detergent Compatible Bradford Assay Kit (Thermo Scientific™, Waltham, MA, USA) was used for measurement of total protein concentration. Unless otherwise mentioned, all other reagents were from Sigma-Aldrich.

### 2.2. Cells and Plasmid Constructs

Huh7.5 [24] and Huh7-Lunet [25] cells were maintained in high-glucose Dulbecco’s modified Eagle medium, supplemented with 10% (*v*/*v*) heat-inactivated foetal calf serum, 2 mM glutamine, 100 U/mL penicillin/streptomycin and 0.1 mM non-essential amino acids.

Plasmids encoding the full-length genome strains HCV-2a (JFH1) [26], HCV-3a (DBN-3a) [27], the Renilla luciferase reporter virus JcR-2a (pFK_I389RLuc2a_Core_3-Jc1) [28], as well as the pFK-I389PI-Luc/NS3-3′/JFH1 subgenomic HCV replicon and its corresponding non-replicating construct, ΔGND [29] (a schematic representation of the constructs is shown in the Section 3), were used for in vitro transcription of viral RNA.

The pLSD1 plasmid contains the human wild-type (wt) LSD1 coding sequence and was constructed via subcloning of the LSD1 XhoI-NotI fragment from the pOZ-N-Flag-HA-LSD1 plasmid, kindly provided by Shi and colleagues [30], into the corresponding sites of pCDNA3.1/myc-His(-) C vector (Invitrogen, Waltham, MA, USA). The plasmid was verified with sequencing.

### 2.3. Generation of Stable Cell Lines and DNA Transient Transfections

The Hu1b stable cell line overexpressing LSD1 was generated via transfection of Huh7.5 cells with 10 μg of pLSD1 expression plasmids, using jetPEI (PolyPlus, Strasbourg, France), according to the manufacturer’s protocol. Similarly, the pCDNA3.1/myc-His(-) C vector was transfected into Huh7.5 cells to provide the “empty vector” control Huh7.5 cell line (denoted as Huh7.5 or pcDNA). Forty-eight hours post transfection (p.t.), transfected cells were selected by addition of 700 μg/mL G418, and individual clones were obtained by limiting dilution. The construction of HCV core (C2-3) and HCV NS5A (NS5A) overexpression cell lines has been reported elsewhere [31,32].

For DNA transfection, Huh7.5 cells were transiently transfected with 3 μg of pLSD1 or pCDNA3.1/myc-His(-) C plasmid DNA using JetPEI^®^ reagent and harvested 48 h p.t. Protein expression was normalised to total protein determined using Bradford assays carried out according to the manufacturer’s instructions.

### 2.4. Viral Infections and RNA Transfections

Viral infections of permissive Huh7.5 cells with the HCV-2a and HCV-3a infectious clones were carried out essentially as previously described [33], at multiplicity of infection (MOI) = 1.

For RNA transfections, the protocol used is essentially described by Lohmann and colleagues [34]. In vitro transcribed RNAs were introduced via electroporation into Hu1b or Huh7.5 cells. Briefly, cells were electroporated with 10 μg RNA and equal amounts of carrier t-RNA (Invitrogen, Waltham, MA, USA). Following transfection, cells were harvested, seeded into 24-well plates and collected at the appropriate time points. Luciferase activity of the cell extracts was determined using the Renilla Luciferase (for full-length RNA) and Firefly Luciferase (for subgenomic replicons) Assay Systems (Promega, Madison, WI, USA). Then, luciferase activity was normalised to protein concentration to yield relative luciferase activity (RLA). All electroporation experiments were carried out 7 times in triplicate.

### 2.5. mRNA Analysis

Total cellular RNA was isolated using NucleoZOL (Macherey-Nagel, Düren, Germany), according to manufacturer’s instructions. Reverse transcription reactions were performed by using 1 μg total RNA, p(dN)_6_ random hexamers (Roche, Basel, Switzerland) and Moloney murine leukemia virus (MMLV) reverse transcriptase (Promega, Madison, WI, USA). Amplification of LSD1, 18S rRNA, NS3-2a and NS3-3a genes was carried out using gene-specific primers (shown in Table S1), Kapa^®^ SYBR Fast Master Mix (Kapa Biosystems, Wilmington, MA, USA) or GoTaq^®^ qPCR Master Mix (Promega, Madison, WI, USA) in a Corbett Rotor Gene 6000 thermocycler (Qiagen, Hilden, Germany). Results were analysed with the internal standard curve method and normalised to the 18S rRNA to provide relative mRNA expression. All experiments were carried out 4 times in triplicate.

### 2.6. Protein Analysis and Immunoprecipitation

Cells were washed twice with PBS and harvested in RIPA buffer (1.0% (*v*/*v*) NP-40, 1% (*w*/*v*) sodium deoxycholate, 0.1% (*w*/*v*) SDS, 25 mM Tris pH 7.6, 150 mM NaCl), supplemented with protease inhibitor cocktail (Roche) and phosphatase inhibitor cocktail (Roche, Basel, Switzerland), to prepare whole cell extracts. Nuclear and cytoplasmic extracts were prepared as described before [35]. Protein concentrations were determined with the Bradford assay. Whole cell (40–50 μg), nuclear (5 μg) or cytoplasmic (20 μg) extracts were resolved in 10–12% (*w*/*v*) SDS-PAGE gels, transferred onto Immobilon-P PVDF membranes (Merck-Millipore, Darmstadt, Germany) and incubated overnight with the following antibodies: β-actin (#3700), LSD1 (#2184), IFITM3 (#59212), p21 (#2947), Histone H3 (#9715), Occludin (#91131) and CD81 (#10037) by Cell Signalling Technology^®^ (Danvers, MA, USA), IFITM3 (ES2592) by ELK Biotechnology (Denver, USA), goat anti-rabbit IgG (#AP132P) and goat anti-mouse (AP124P) by Millipore. Chemiluminescence was detected using Immobilon Crescendo HRP Substrate (Merck-Millipore, Darmstadt, Germany). The rabbit polyclonal anti-HCV core and anti-HCV NS5A antibodies were constructed in our laboratory, as described in [36,37]. Protein expression was normalised to β-actin or histone H3.

Quantity One 4.4.1 densitometry software (Bio-Rad, Hercules, CA, USA) was used for protein band quantification. Blot photographs presented herein are representative of samples that were electrophoresed together and processed in parallel.

For immunoprecipitation experiments, at least 150 μg of whole cell extracts from Huh7.5 and Hu1b cells were incubated on a rotary shaker at 4 °C overnight, with the anti-IFITM3 antibody. Resulting complexes were precipitated with protein A-agarose beads (Santa Cruz, TX, USA), followed by three washes with lysis buffer (50 mM Tris pH 8, 150 mM NaCl, 0.02% (*w*/*v*) sodium azide, 0.1% (*w*/*v*) SDS, 100 μg/mL PMSF, 1% (*v*/*v*) NP-40, 0.5% (*w*/*v*) sodium deoxycholate), separated on a 12% (*w*/*v*) SDS-PAGE gel, transferred onto PVDF membranes and, finally, blotted with anti-LSD1 and anti-Mono-Methyl Lysine (#14679, Cell Signalling Technology) antibodies. In this case, the light chain IgG (IgG-L) band was used as internal control.

All protein expression experiments were performed at least in triplicate.

### 2.7. LSD1 Knockdown and Pharmacological Inhibition Studies

For siRNA-mediated knockdown of LSD1 mRNA, Huh7.5 cells were seeded in 24-well plates at 40% confluence and treated with 8 μM siRNA oligonucleotides against LSD1 (siLSD1) or negative non-silencing control (siNC) (Qiagen, Hilden, Germany) using DharmaFECT 2 transfection reagent, (Dharmacon RNA Technologies, Lafayette, CO, USA). Forty-eight hours p.t., the degree of LSD1 silencing was measured using RT-qPCR and Western blotting, as described in the mRNA and protein analysis sections.

As for the pharmacological inhibition of LSD1 enzymatic activity, Huh7.5 cells were treated with the specific LSD1 inhibitor Bizine (Cayman Chemical, Ann Arbor, MI, USA) at a concentration of 5 μM for 48 h prior to HCV or mock infection. The use of trypan blue exclusion assay showed that the levels of dead cells were kept at less than 5% of the total cell number throughout the inhibition experiment. LSD1 activity inhibition was assessed by determination of the LSD1 downstream target p21 protein expression levels [38], via immunoblotting.

Both experiments were carried out at least 8 times in triplicate.

### 2.8. LSD1 Activity Assay

LSD1 enzymatic activity was measured in whole cell extracts with an LSD1 Inhibitor Screening Assay Kit (Cayman Chemicals, Ann Arbor, MI, USA), according to the manufacturer’s instructions. All protein samples were diluted 1 to 10 before being placed into the wells. RIPA buffer was added to the background wells and LSD1 peptide provided in the kit was used as a positive control. The results were normalised to total protein concentration and analysed as percentage change of the control cell lysates that were arbitrarily set as 1. The experiment was carried out twice in duplicate.

### 2.9. Immunofluorescence

Immunofluorescence analysis was performed to determine LSD1 localisation, using an anti-LSD1 antibody, as previously described [39].

### 2.10. Statistical Analysis

Statistical analysis was performed using Student’s *t*-test. For comparisons between three or more groups analysis of variance (ANOVA) was employed, followed by post hoc pairwise testing with Student’s *t*-test, with *p* ≤ 0.05 considered statistically significant (* *p*-value ≤ 0.05, ** *p*-value ≤ 0.005, *** *p*-value ≤ 0.001). Unless otherwise shown, statistical analysis was carried out between mock and infected samples.

## 3. Results

### 3.1. HCV Infection Modulates LSD1 Expression and Activity

**Figure 1:**
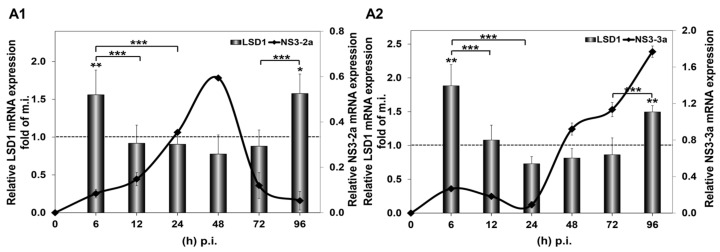
HCV-mediated regulation of LSD1 mRNA in Huh7.5 cells. The histograms depict relative LSD1 (bars) and HCV NS3 (black line) mRNA expression during infection with HCV-2a (**A1**) and HCV-3a (**A2**) laboratory clones, compared to mock infection. Total RNA was extracted from Huh7.5 cells and subjected to RT-qPCR. 18S rRNA was used as an internal control. Values were calculated as fold of the respective mock-infected controls that were arbitrarily set as 1 (represented by the dashed line). (* *p*-value ≤ 0.05, ** *p*-value ≤ 0.005, *** *p*-value ≤ 0.005).

In order to study the impact of in vitro HCV infection on LSD1 expression, Huh7.5 cells were infected with two different HCV genotypes, HCV-2a and HCV-3a, as described above. Infected cells were harvested from 6 to 96 h p.i. and used for RNA extraction and whole cell extract preparation. RNA extracts were subjected to RT-qPCR, while whole cell extracts were used for Western blotting, as well as for LSD1 activity measurements. As shown in Figure 1, infection with both HCV genotypes up-regulated LSD1 mRNA levels by more than 1.5-fold at 6 h and 96 h p.i., as compared with mock-infected cells. Remarkably, the sharp increase at the beginning of infection reverted to background levels until the 96 h time point. These results were in stark difference with the constitutive LSD1 mRNA expression, which followed a pattern of gradual increase up to 2-fold at 48 h and then dropped to 0.5-fold at 96 h, when compared with the 6 h mock-infected control value (Figure S1A).

**Figure 2:**
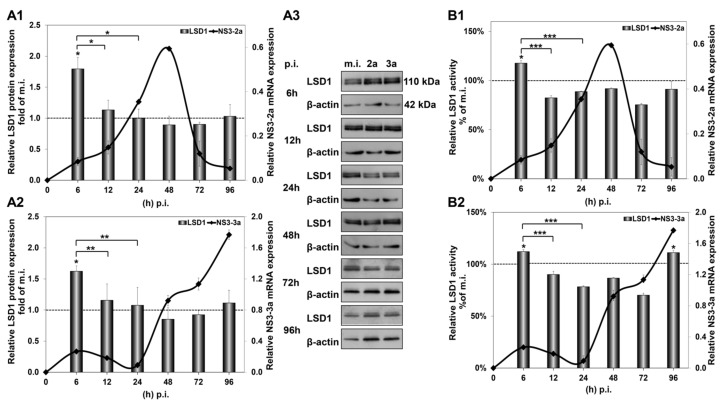
HCV-mediated regulation of LSD1 protein expression and activity in Huh7.5 cells. The histograms depict the relative LSD1 protein expression during HCV infection with HCV-2a (**A1**) and HCV-3a (**A2**). Whole cell extracts from the infection experiments were used in immunoblotting analysis (**A3**), and β-actin was the loading control. The blots were subjected to densitometry and plotted in expression histograms. Values were calculated as fold of the respective mock-infected controls that were arbitrarily set as 1 (dashed line). LSD1 activity during HCV-2a (**B1**) and HCV-3a (**B2**) infection was measured in whole cell extracts and normalised to total protein to provide relative activity. All values were calculated as a percentage of the respective mock-infected controls (dashed line) that were arbitrarily set as 1. Relative HCV NS3 mRNA expression was used to monitor HCV infection (black line). (* *p*-value ≤ 0.05, ** *p*-value ≤ 0.005, *** *p*-value ≤ 0.001).

The significant increase in LSD1 mRNA at 6 h p.i. was accompanied by a similar increase in LSD1 protein expression, shown in Figure 2(A1,A2), that was not observed in mock-infected cells (Figure S1B). Importantly, LSD1 enzymatic activity was also found to be increased by 20% at the same time point, shown in Figure 2(B1,B2), when constitutive LSD1 activity was observed to be down-regulated by at least 50% from 24 h to 96 h in comparison with the 6 h mock-infected control (Figure S1C). Interestingly, protein expression was maintained at background levels until 96 h p.i., despite the increase in mRNA observed at the same time point.

Taken together, our results show that not only does HCV infection tightly regulate the gene expression and enzymatic activity of LSD1, but these changes occur regardless of viral genotype.

### 3.2. LSD1 Expression Is Modulated by Specific HCV Viral Proteins

**Figure 3:**
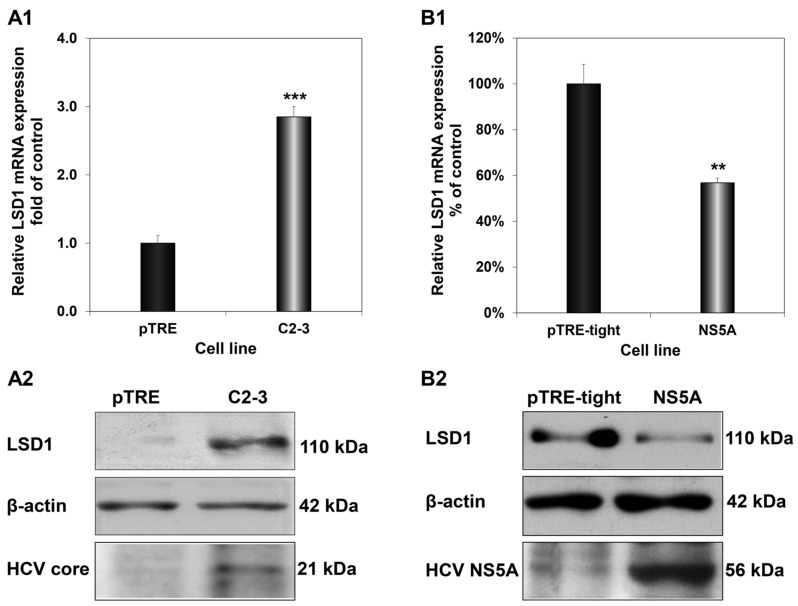
Differential regulation of LSD1 by HCV core and HCV NS5A. LSD1 mRNA (**A1**) and protein (**A2**) levels were measured in the C2-3 hepatic cell line, which stably expresses HCV core. Similar mRNA (**B1**) and protein (**B2**) expression measurements were carried out in an HCV NS5A-expressing hepatic cell line (NS5A). Endogenous LSD1 mRNA levels were measured using RT-qPCR with 18S rRNA as internal control. Expression values of the control pTRE and Huh pTRE-tight cell lines (black bars) were arbitrarily set as 1-fold or 100%, and the values in the viral protein expressing cells were calculated as fold or percentage of these. LSD1, HCV core and HCV NS5A protein levels were determined with immunoblotting and β-actin was used as a loading control. (** *p*-value ≤ 0.005, *** *p*-value ≤ 0.001).

HCV exerts its regulatory action on the host mainly through the multifunctional viral protein HCV core, which is one of the first proteins encountered by the host cell, and HCV NS5A, whose levels build up in the infected cell later, upon viral translation [40]. Previous studies from our laboratory have shown that these proteins tend to antagonise each other’s function to achieve fine-tuning of key host genes that could be vital for the completion of the viral life cycle [31,41]. To determine whether this is the case with LSD1, we examined the impact of HCV core and HCV NS5A on LSD1 expression, by measuring LSD1 mRNA and protein levels in two cell lines, stably expressing HCV core (C2-3) and HCV NS5A (NS5A), compared to their control cells. Figure 3(A1,A2) show that HCV core up-regulated both LSD1 mRNA and protein expression significantly. On the contrary, overexpression of HCV NS5A decreased LSD1 mRNA and protein levels by half (Figure 3(B1,B2)). Overall, our data showed that LSD1 transcription was differentially regulated by HCV core and HCV NS5A, as originally hypothesised.

### 3.3. HCV Propagation Is Highly Dependent on LSD1 Endogenous Levels

#### 3.3.1. LSD1 Overexpression Inhibits HCV Infection

The tightly controlled temporal regulation of LSD1 expression and activity throughout the life cycle of HCV points to the importance of this gene to the successful establishment of HCV infection. Therefore, we used genetic engineering approaches to assess the effect of both high and low endogenous LSD1 levels on HCV propagation.

**Figure 4:**
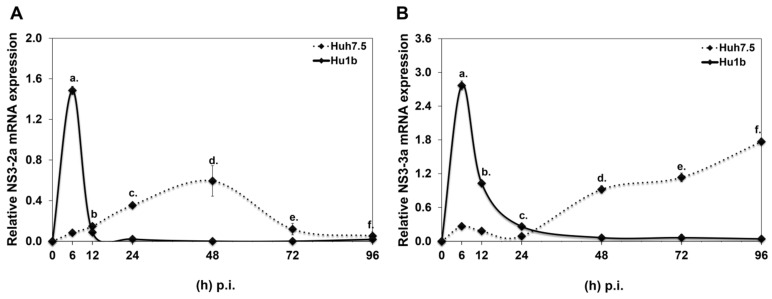
HCV RNA replication in LSD1 overexpression. Hu1b (bold line) and Huh7.5 (dashed line) were infected with (**A**) HCV-2a and (**B**) HCV-3a. Relative HCV NS3 mRNA expression at various time points p.i. was used to monitor HCV active replication. (stats key: (**A**)a: ***, (**A**)b: n.s., (**A**)c: ***, (**A**)d: ***, (**A**)e: **, (**A**)f: *. (**B**)a: ***, (**B**)b: ***, (**B**)c: n.s., (**B**)d:***, (**B**)e: ***, (**B**)f: *** with * *p*-value ≤ 0.05, ** *p*-value ≤ 0.005, *** *p*-value ≤ 0.001 and n.s.: not significant).

Initially, an Huh7.5-based cell line that stably overexpresses LSD1 (Hu1b) was constructed and characterised, as shown in Figure S2. Compared with the “empty vector” control cells (Huh7.5), the Hu1b clone was found to overexpress LSD1 mRNA by 5-fold (Figure S2A1), protein by 2-fold (Figure S2A2) and possessed twice the enzymatic activity of the control cells (Figure S2A3). After testing the constitutive LSD1 expression levels between naïve Huh7.5 and pCDNA3.1 cells, we found them to be similar and continued experimentation with Huh7.5 cells as the control cell line for subsequent infection time courses with HCV-2a and HCV-3a. Figure 4 demonstrates HCV-2a and HCV-3a rates of active replication [42] in both cell lines, as portrayed by the continuous mRNA expression of the HCV NS3 gene. To our surprise, replication of both viruses was shown to be completely blocked by the presence of increased LSD1 levels; after a short time, there was an HCV NS3 expression boost of at least 17-fold for HCV-2a and 10-fold for HCV-3a, occurring within a time frame dependent on the specific viral strain. Similar results were obtained from cells transfected with pLSD1 30 h prior to infection with HCV-3a (Figure S3).

The progression of infection was evaluated using HCV core levels in the Hu1b clone versus control cells and, as revealed by immunoblotting analysis, the protein levels of HCV core diminished, suggesting that HCV infection was blocked (Figure S4).

**Figure 5:**
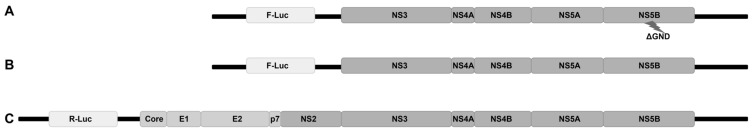
Schematic representation of HCV genome constructs used for electroporation of Huh7.5 and Hu1b cell lines, as described in Section 2.3. (**A**) Replication-deficient HCV replicon, ΔGND; (**B**) replication-efficient HCV replicon, pFK-I389PI-Luc/NS3-3′/JFH1; and (**C**) full HCV genome, JcR-2a.

Given that by the end of 24 h p.i., HCV has completed most of its viral life cycle steps [43], this violent blockage of HCV propagation warranted further investigation into the early steps of the viral life cycle that could have been hindered in the Hu1b cells. To clarify this point, we used three HCV-2a RNA constructs, shown in Figure 5, to break down the early part of the viral life cycle into segments. Indeed, the ΔGND deficient subgenomic replicon depicted in Figure 5A contains a mutant NS5B RNA-dependent RNA polymerase that does not allow replication of the translated RNA and was used to monitor viral translation. Its respective wt pFK-I389PI-Luc/NS3-3′/JFH1 subgenomic replicon (Figure 5B) is replication-competent, but still lacks all the structural regions of the virus, enabling us to evaluate the role of HCV core in the LSD1-mediated modulation of HCV replication and absence of capsid formation. Finally, in order to assess the putative effect of LSD1 on the initial steps of viral infection, we electroporated the full-length viral genome (Figure 5C) into the cells, thereby bypassing HCV entry and endocytosis.

**Figure 6:**
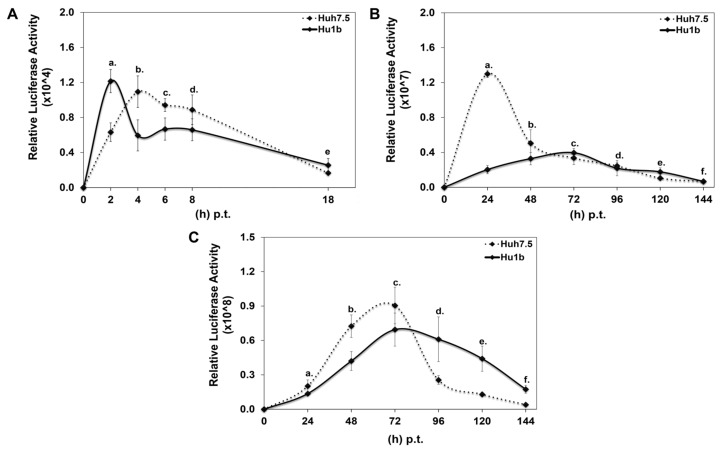
Effect of LSD1 overexpression on HCV life cycle. (**A**) Huh7.5 and Hu1b cell lines were electroporated with an HCV replication-deficient subgenomic replicon. Luciferase activity values were normalised to total protein to give relative luciferase activity, which was used to monitor HCV translation. (stats key: (**A**)a: *, (**A**)b: *, (**A**)c: *, (**A**)d: n.s., (**A**)e: n.s.) (**B**) Huh7.5 and Hu1b cell lines were electroporated with an HCV replication-efficient subgenomic replicon. Relative luciferase activity, calculated as described in (**A**), was used to monitor HCV replication. (stats key: (**B**)a: ***, (**B**)b: *, (**B**)c: *, (**B**)d: n.s., (**B**)e: *, (**B**)f: n.s.) (**C**) Huh7.5 and Hu1b cell lines were electroporated with the whole HCV genome. Relative luciferase activity, calculated as described in (**A**), was used to monitor HCV replication. (stats key: (**C**)a: **, (**C**)b: ***, (**C**)c: *, (**C**)d: ***, (**C**)e: ***, (**C**)f: ***, with * *p*-value ≤ 0.05, ** *p*-value ≤ 0.005, *** *p*-value ≤ 0.001 and n.s.: not significant).

Each DNA construct was subjected to in vitro transcription, and the produced RNA was electroporated into Hu1b and control Huh7.5 cells together with equal amounts of carrier tRNA. Cells were harvested at specific time intervals, depending on whether HCV translation or replication would be examined, and subjected to luciferase assays. Figure 6A shows that HCV translation occurred 2 h faster in the LSD1 overexpressing cell line, relative to the control; however, after that initial boost, a significant reduction was observed in the viral translation rate between Hu1b and Huh7.5 cells. Furthermore, replication of the wt subgenomic replicon was dramatically reduced at 24 h p.t., at which time point the corresponding replication trend in Huh7.5 cells reached its peak (Figure 6B). Moreover, the absence of HCV core intensified the attenuating effect of LSD1 on HCV replication. Surprisingly, though, electroporation of the full-length HCV genome in Hu1b cells was not enough to block viral propagation as observed after natural infection in Figure 4, but it did stall it significantly (Figure 6C). The later observation suggested that viral entry and/or endocytosis may be necessary for the LSD1-mediated inhibition of HCV infection, at least in vitro.

#### 3.3.2. Down-Regulation of LSD1 Affects HCV Propagation

**Figure 7:**
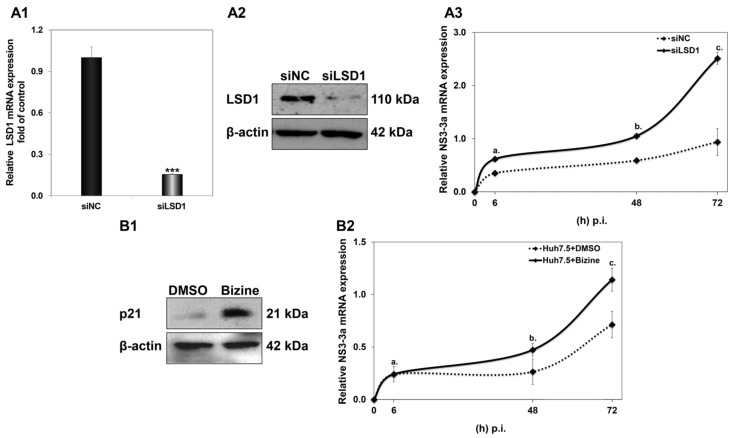
Effect of LSD1 knockdown on LSD1 endogenous expression. (**A1**) Relative LSD1 mRNA expression in siLSD1-transfected Huh7.5 cells, compared to negative control (siNC). (**A2**) Representative immunoblot of LSD1 expression in siLSD1-transfected Huh7.5 cells and negative control (siNC). (**A3**) Huh7.5 cells were infected with HCV-3a 48 h after treatment with siLSD1 RNA oligonucleotides. Relative HCV NS3 mRNA in siLSD1 (bold line) and scrambled (siNC) siRNA (dashed line) was used to monitor HCV active replication. (stats key: (**A**)a: *, (**A**)b: ***, (**A**)c: *** with * *p*-value ≤ 0.05, *** *p*-value ≤ 0.001). (**B1**) Representative immunoblot of p21, a downstream target of LSD1, 48 h post treatment with Bizine or DMSO. (**B2**) Huh7.5 cells were infected with HCV-3a 48 h after treatment with pharmacological inhibitor Bizine (bold line) or DMSO as control (dashed line). Relative HCV NS3 mRNA was used to monitor HCV active replication. (stats key: (**B**)a: n.s., (**B**)b: **, (**B**)c: ** with ** *p*-value ≤ 0.005, n.s.: not significant).

Having confirmed that LSD1 overexpression could be detrimental to the establishment of HCV infection, despite an apparent sharp boost at the very early part of the viral life cycle, we carried on with the opposite approach by reducing LSD1 endogenous levels and activity before HCV infection. Firstly, siRNA knockdown experiments were performed using a pair of specific siRNA oligonucleotides against LSD1 that were introduced in Huh7.5 cells 48 h prior to infection. Scrambled siRNA oligonucleotides were used as negative control (siNC) at the same concentration of 8 nM. The results shown in Figure 7(A1,A2) demonstrate a knockdown efficiency of at least 80% for both RNA and protein expression of LSD1 in Huh7.5 cells. We also established that compared with naïve cells, siNC did not produce any off-target effects at the concentration used.

Next, the transfected cells were infected with HCV-3a; harvested at 6 h, 48 h and 72 h p.i.; and subjected to total RNA isolation and RT-qPCR with NS3-3a primers, so that HCV replication could be monitored. As previously observed by our group [44], HCV-3a requires a longer time to reach measurable replication levels than the HCV-2a strain. Thus, we hypothesised that HCV-3a would make a better model for evaluating putative LSD1 knockdown-mediated effects on the very early steps of the viral life cycle. As shown in Figure 7A3, LSD1 knockdown greatly facilitated HCV replication compared with the scrambled control, achieving a significant two-fold increase, even at 48 h p.i., where NS3 expression has been shown to be relatively low (see Figure 4B). Notably, at the end of this time course at 72 h, we observed that the HCV-3a replication rate was elevated by at least 2.5-fold, compared with cells with normal endogenous LSD1 levels.

A second line of validation for the effect of low LSD1 levels on HCV infection was pursued by pre-treating Huh7.5 cells with the pharmacological inhibitor Bizine for 48 h prior to infection. Bizine is a phenelzine analogue which selectively affects LSD1 activity [45]. The solvent of Bizine, DMSO, was used as vehicle control. Figure 7B2 shows that inhibition of LSD1 catalytic activity was enough to significantly enhance the HCV-3a replication rate, leading to a 1.6-fold increase at 72 h. Furthermore, for validation purposes, we determined the expression levels of p21, which, as a downstream target of LSD1 [38], should be increased in the presence of Bizine (Figure 7B1). This was indeed observed in our experiments.

Taken together, the results described in paragraph *3.3* suggested that the overexpression of LSD1 exerted an inhibitory effect on the establishment of successful HCV infection, which was alleviated upon LSD1 knockdown.

### 3.4. Effect of LSD1 on Selected HCV Entry Receptors

**Figure 8:**
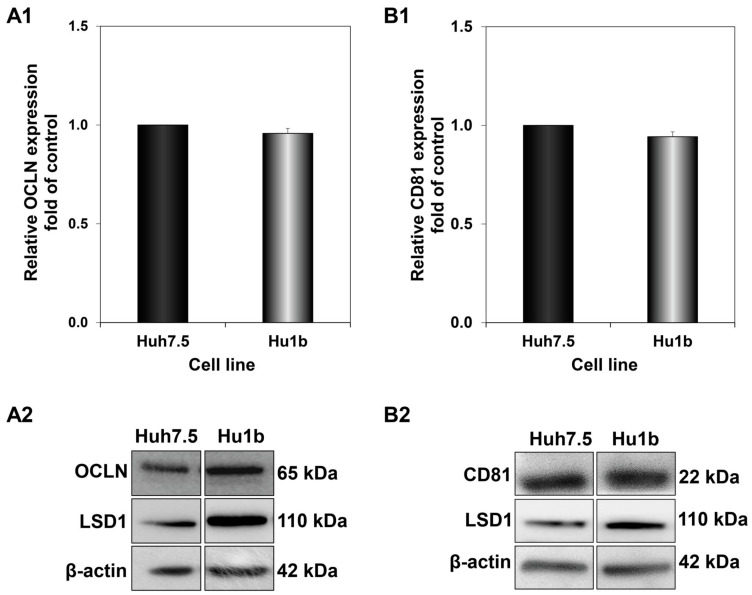
Effect of LSD1 overexpression on host cell receptors implicated in HCV entry. Blots were subjected to densitometry and plotted in expression histograms representing the relative OCLN (**A1**) or CD81 (**B1**) relative expression over β-actin loading control. Expression values of the control (black bar) were arbitrarily set as 1-fold and the values in Hu1b cells were calculated as fold of this. Representative immunoblot of OCLN (**A2**) and CD81 (**B2**) protein expression in LSD1-expressing cells and controls.

The results obtained so far indicated that fine-tuning of LSD1 expression levels at different steps of the viral life cycle is crucial for successful HCV infection. Additionally, LSD1 overexpression has a notable effect on the first steps of infection, focusing on virus entry. It has been suggested that HCV entry is a five-step process, composed of virus attachment, translocation, endocytosis, translocation/fusion and uncoating [46]. In order to study the impact of LSD1 on HCV entry, we prepared whole cell extracts from Huh7.5 and Hu1b cells and subjected them to Western blotting with antibodies against two molecules that have been proven to be vital for HCV entry, CD81 and occludin (OCLN). CD81 is a cell surface protein and a member of the tetraspanin family that binds the HCV E2 envelope glycoprotein after the attachment of the virus onto the membrane [47]. OCLN, a tight junction protein, acts later in HCV entry by facilitating its internalisation [48]. However, as shown in Figure 8, there was no significant change observed in CD81 or OCLN expression in Hu1b, compared to Huh7.5 control cells. Similar results were obtained after HCV-3a infection of both cell lines.

### 3.5. LSD1 Inhibits HCV Entry by Modulating the Activity of HCV Endocytic Pathway Component IFITM3

Next, we carried on to study endocytosis, a later phase of HCV entry, where the endocytic trafficking of virus-containing endosomes takes place. HCV particles have to circumvent lysosomal entrapment and degradation in order to achieve infection of host cells. Interferon-induced transmembrane protein 3 (IFITM3), a protein mainly located on endosomal and lysosomal membranes, exerts antiviral activity against enveloped viruses that enter cells through either pH-dependent or pH-independent fusion with endosomal compartments [49]. It has been previously reported that LSD1 demethylates K88-Lys of IFITM3, regulating its antiviral activity [12]. We theorised that LSD1 could inhibit HCV propagation through the regulation of IFITM3 methylation status. To achieve this, LSD1, best known as a nuclear protein, would have to be able to shuttle between the nucleus and cytoplasm and reach these compartments located in the proximity of the plasma membrane. In our hands, this was indeed the case, as can be observed in immunofluorescence experiments that clearly verified the presence of LSD1 in both the cytoplasm and nucleus of Huh7.5 cells (Figure S5A). In addition, we performed fractionation experiments that demonstrated the presence of LSD1 in cytoplasmic and nuclear extracts analysed with Western blotting (Figure S5B).

IFITM3 is an interferon-stimulated gene [50]. However, Huh7.5 cells are known to be defective in interferon signalling [51], a trait that shapes their permissiveness to HCV infection. Thus, we examined whether IFN-β treatment for 24 h could enhance IFITM3 expression levels in our cells.

As shown in Figure S6, IFITM3 was expressed in both cell lines, albeit at least 40% lower on average in Hu1b than in Huh7.5 control cells. Evidently, IFITM3 expression was indeed up-regulated by IFN-β in both cell lines, suggesting that at least some parts of interferon signalling are functional in Huh7.5 cells.

**Figure 9:**
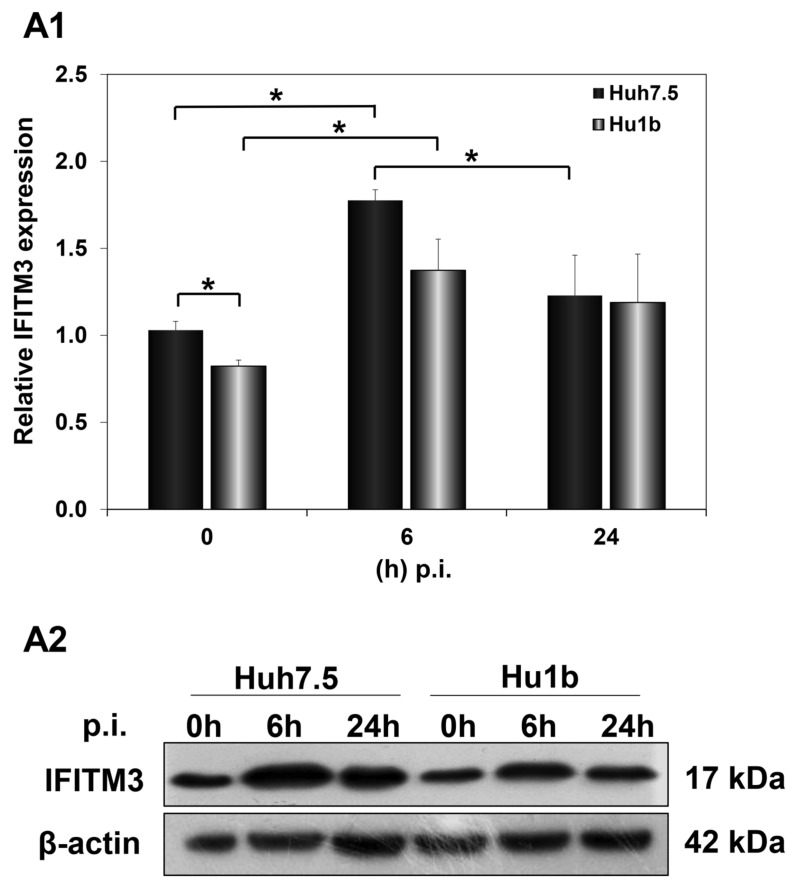
IFITM3 protein expression in HCV infection in vitro. Whole cell extracts of HCV-3a-infected cells were used in immunoblotting analysis and β-actin was the loading control. (**A1**) The graph depicts the relative IFITM3 expression (as IFITM3 over β-actin ratio) at various time points after in vitro HCV infection of Huh7.5 and Hu1b cell lines. (**A2**) A representative immunoblot that was subjected to densitometry and plotted in expression histograms, as shown in (**A1**). (* *p*-value ≤ 0.05).

Subsequently, we examined the effect of HCV infection on IFITM3 protein expression levels in the first 24 h p.i., when LSD1 almost completely inhibits viral propagation. Huh7.5 and Hu1b cells were infected with HCV-3a at MOI = 1 and harvested at 0 h, 6 h and 24 h p.i. In Figure 9(A1,A2), IFITM3 protein expression was shown to be up-regulated at 6 h post HCV-3a infection in both Huh7.5 and Hu1b cells, compared with non-infected cells (0 h), rendering IFITM3 a part of the host immune response mechanism against HCV entry. IFITM3 expression receded to background levels only in the control cells by 24 h. The LSD1-mediated decrease in IFITM3 expression seen in naïve cells in the presence of IFN-β was not observed during infection. Interestingly, when we measured IFITM3 protein expression under LSD1 knockdown conditions in Huh7.5 cells, IFITM3 was increased (Figure S7). This result provides further evidence that LSD1 is a negative regulator of IFITM3 protein expression.

**Figure 10:**
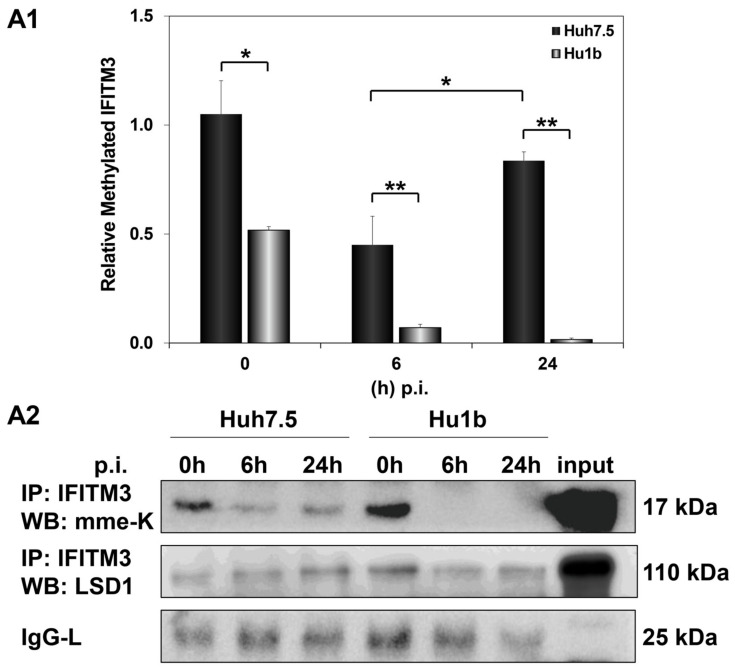
LSD1 activates IFITM3 via regulation of its monomethylation status in HCV infection. (**A1**) The graph depicts the relative expression of mme-K IFITM3 (ratio of mme-K IFITM3 over IgG-L expression), which represents the inactive fraction of the IFITM3 protein, after HCV-3a infection of Huh7.5 and Hu1b cell lines. (**A2**) Representative Western blot image of whole cell extracts from HCV-3a-infected Huh7.5 and Hu1b cells co-immunoprecipitated with total IFITM3 and immunoblotted with mme-K and LSD1 antibodies. The blot was subjected to densitometry and plotted in expression histograms, as shown in (**A1**). IgG-L was used as loading control. (* *p*-value ≤ 0.05, ** *p*-value ≤ 0.005).

We then investigated whether LSD1-driven regulation of IFITM3 enzymatic activity could be responsible for the failed establishment of the HCV life cycle in Hu1b cells. Monomethylation of IFITM3 at Lys-88 residue (mme-K) is known to block its homodimerization and bridging of late endosomes with lysosomes [49]. Thus, we immunoprecipitated IFITM3 from mock- and HCV-infected Huh7.5 and Hu1b whole cell lysates to assess its methylation status in the presence of LSD1. The IFITM3 immunoprecipitated lysates were subjected to Western blotting with antibodies able to detect the monomethylated/inactive form of IFITM3, as well as LSD1 itself. As shown in Figure 10, mme-K IFITM3 decreased at 6 h p.i. in both cell lines. However, the mme-K IFITM3 was dramatically reduced in Hu1b, almost abolished by 24 h, suggesting that overexpression of LSD1 in early HCV infection activated IFITM3 through demethylation. Conversely, HCV was able to bypass IFITM3 activation and increase the inactive IFITM3 fraction in Huh7.5 cells at 24 h p.i. Notably, co-immunoprecipitation of LSD1 with the mme-K IFITM3 form strongly supported the presence of LSD1 in the precipitated immunocomplexes together with IFITM3 in both cell lines.

## 4. Discussion

Recently, the epigenetic controller LSD1 has emerged as a key molecule for the completion of the life cycle of many viruses, as attested by the numerous examples given in the Introduction section. So far, there has not been any research to suggest direct LSD1 involvement in the establishment of HCV infection, even though it has recently been shown that HCV-induced epigenetic alterations persist even after DAA treatment. Changes in the expression of liver proteins caused by HCV and hepatocyte-specific epigenetic modifications are linked to the development of HCC [52,53], making the discovery of novel pro- or anti-HCV host factors even more pertinent to the fight against HCC in chronic HCV patients, cured or not.

In this study, we showed for the first time that HCV infection transcriptionally regulated LSD1, correspondingly affecting protein and enzymatic activity levels. Although these changes occurred independently of HCV genotype, they followed a specific temporal pattern, occurring at the beginning and the end of the experiment. However, protein expression of LSD1 did not follow the 96 h p.i. mRNA increase, suggesting a possible lag response of endogenous LSD1 expression towards the late hours. The effect was very clear for the HCV-2a virus, which is known to have a 4-day replication cycle in vitro [54], but it was also observed for HCV-3a, which grows more slowly and follows a different prolonged replication pattern, exhibiting a lag phase before its replication initiation. Only recently has it been shown that these two viral strains follow different kinetics, possibly due to significant differences between their NS5A functions [55]. In addition, previous infectivity measurements from our laboratory showed that HCV-3a releases viral particles from hepatocytes days later compared with HCV-2a [44].

In spite of being an RNA virus and having a cytoplasmic life cycle, HCV alters gene expression to disrupt or enhance different cellular processes and mechanisms, including epigenetics [56], to rearrange the host cell microenvironment in a beneficial way for its propagation. In particular, HCV is known to regulate cell proliferation/the cell cycle by interfering with host regulators of different phases of the cell cycle, such as cyclin kinase complexes [57]. Other examples are the modulation of apoptosis through caspase-3-dependent or -independent pathways [58] and the enhancement of autophagy as a means of blocking stress-induced host immune responses [59]. Finally, the HCV life cycle is highly dependent on host cell metabolism, mainly lipid metabolism, for particle composition, entry, replication and assembly [60,61], as well as immune escape [62]. LSD1 is involved in the epigenetic regulation of mediators of all these crucial processes [63,64,65,66]. Therefore, it seems that HCV-mediated manipulation of LSD1 functions to coordinate different cellular immunometabolic pathways, so that the virus may establish a favourable environment for viral propagation, causing severe dysfunction as a result.

Looking into the viral mechanism of action, we observed that the multi-functional HCV proteins, core and NS5A, were responsible for the differential regulation of LSD1, whereby HCV core increased and NS5A decreased LSD1 expression. The HCV core protein is the first structural protein encountered by the cell upon viral entry and produced during HCV translation [67], which could partially explain LSD1 up-regulation in the earliest steps of HCV infection. On the other hand, NS5A probably exerts its actions after being produced and may help to restore LSD1 expression to its pre-infection levels. This is not the first time such an antagonistic action is reported for these specific viral proteins. Previous studies from our laboratory have shown that the iron-regulatory hormone hepcidin is also differentially regulated in the same way and is crucial for the establishment of successful infection [32]. p53 and c-Jun regulation is also a result of HCV core and NS5A antagonistic functions [68,69,70,71]. The tight temporal control of HCV on LSD1 expression left us speculating on the role of LSD1 endogenous levels on the consecutive steps of HCV entry and virus life cycle. This question was addressed by LSD1 overexpression and knockdown experiments in hepatoma cells that were used in in vitro infections. Surprisingly, infection of an LSD1 overexpressing cell line with HCV revealed a strong inhibition of viral propagation, despite a transient sharp peak in the rate of active HCV replication in the first 6 h p.i. This result hinted at a putative inhibiting role for LSD1 on HCV infection, which could explain the need for NS5A to mediate the down-regulation of endogenous LSD1 post endocytosis.

The HCV life cycle has been well studied since the virus was first cultured in vitro almost two decades ago [72]. The viral life cycle stages can be summarised in three main parts: the entry of HCV particles, the translation and replication of HCV RNA and, finally, the assembly and secretion of HCV virions [73]. HCV constructs expressing a replication-efficient subgenomic replicon, its non-replicating analogue and the full-length HCV genome were used to dissect the HCV life cycle steps. This line of experiments revealed that LSD1 overexpression led to faster viral RNA translation and a lower rate of replication in the absence of the HCV structural region. HCV replicon systems have already been proven as valuable tools for the study of the HCV life cycle and their application in research has been critical for the development of DAAs [74]. In this case, their use provided further evidence that the absence of HCV core enhanced the LSD1-orchestrated reduction in the rate of HCV replication. Recently, it has been shown that global translation is regulated epigenetically. For example, methyltransferase SET7/9 catalyses the methylation of the ribosomal component eL42, thus promoting global translation [75]. Furthermore, the sub-cellular localisation of a ribosomal component that affects the ribosomal translation efficiency, Rpl29, is regulated by SET7/9 methylation and LSD1 demethylation on Lys5 [76]. Interestingly, viruses can manipulate the function of such proteins so that they can promote the formation of alternative ribosome complexes and the selection of viral RNA translation over the cellular mRNA [77]. Internal Ribosome Entry Site (IRES)-dependent translation is a common strategy for viruses that need to bypass the host translation machinery requirement for 5′-cap in order to initiate viral translation. One such example is Foot-and-Mouth Disease Virus (FMDV), known to recruit helicase DDX3 and ribosomal component Rpl13 for viral translation initiation [78].

LSD1 is known to modulate lipid metabolism through sterol regulatory element-binding protein (SREBP)-mediated gene expression [79], but when overexpressed it was shown to down-regulate intracellular triglycerides. HCV heavily depends on lipid droplets for its replication and initiates de novo fatty acid synthesis upon infection, as well [80]. Unpublished data from our laboratory have shown that LSD1 overexpression reduced lipid droplets in size and number. Therefore, the lower rate of HCV replication observed in our experiments could be attributed to such a lipid-related mechanism.

An alternative or complementary mechanism to the observed reduced replication rate could be related to capsid entry/endocytosis. To test this notion, we performed electroporation of in vitro transcribed full genome RNAs in hepatoma cells in the presence of LSD1. This resulted in a small but significant delay in the HCV replication rate, which verified and highlighted the importance of these initials steps in the LSD1 effect.

In line with the aforementioned, we evaluated the putative effect of LSD1 overexpression on the expression of specific cell surface receptors known to be implicated in HCV entry, such as CD81 and OCLN, but we did not observe any such changes. Entry is highly regulated and coordinated by a number of cellular and viral factors, occurring after binding of the viral particle to a surface receptor [39]. The latest research showed that LSD1 increases SARS-CoV-2 infection by enhancing its interaction with Angiotensin-converting enzyme (ACE2), the host cellular receptor responsible for SARS-CoV-2 entry [81]. Other host factors that possess crucial roles in the early steps of viral infections have been shown to be prone to demethylation and other post-translational modifications, including IFITM1 and the serine-incorporators (SERINCs) [82].

siRNA against LSD1 and its pharmacological inhibitor Bizine were used to disrupt endogenous expression and activity of LSD1, thereby verifying the detrimental effect of unchecked LSD1 levels on the progression of HCV infection. Similarly, SP-2509 blocked HSV-1 infection [9], while pargyline repressed HBV transcription and replication [10]. Of course, it must be noted that treatment with specific LSD1 inhibitors may also favour some viruses, such as IAV, whose infection was accelerated after treatment with the LSD1 inhibitor trancylpromine [12].

**Figure 11:**
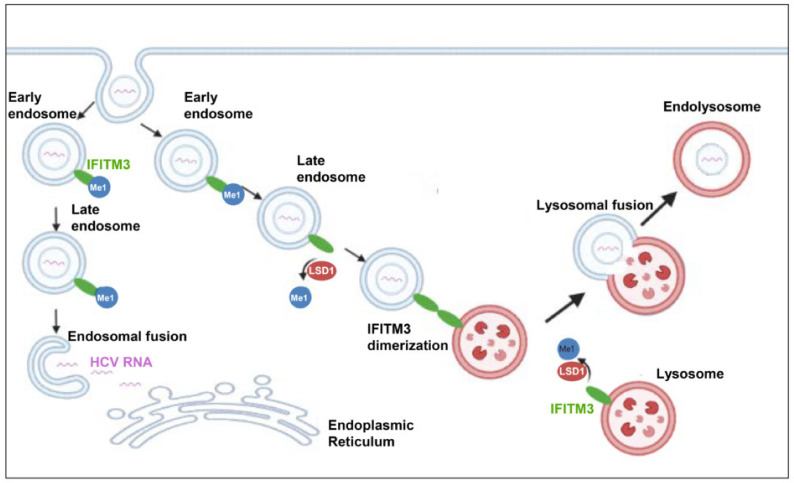
Schematic illustration of LSD1-mediated antiviral pathway. IFITM3 is primarily located on the endosomal and lysosomal membranes in mono-methylated form. When HCV enters the hepatocyte, the viral envelope fuses with the endosomal membrane, the viral RNA is released into the cytoplasm, translated by the host translation machinery to the viral polyprotein, and replication is initiated. Demethylation of IFITM3 by LSD1 triggers the dimerization of IFITM3. Those IFITM3 dimers form clusters that lead to fusion of the endosomal membranes with the lysosomal membranes and, thus, the incoming virus is degraded by the lysosomal enzymes.

Different studies have revealed a variety of endocytic routes for HCV enveloped viral capsids. Following receptor-mediated endocytosis, the viral particles are internalised in a clathrin-mediated way into endosomes, where pH triggers fusion between the viral envelope and endosomal membranes [39]. In order to study whether endocytosis was involved in LSD1-mediated inhibition of HCV propagation, we focused on the endolysosomal protein IFITM3. IFITM3 was up-regulated during the early hours (6 h p.i.) of HCV infection both in overexpressed and normal endogenous LSD1 levels, which suggests that IFITM3 functions as part of the IFN-induced host cell immune response against virus entry. Remarkably, as infection progressed (24 h p.i.), IFITM3 remained increased only in LSD1-overexpressing cells. We realised that in the presence of the virus, LSD1 overexpression strongly demethylated IFITM3, thereby increasing its active form, forcing the shutdown of HCV propagation. Otherwise, upon normal LSD1 endogenous levels, the virus was able to overcome the IFN-LSD1-IFITM3 axis, so that HCV RNA could be released from late endosomes escaping lysosomal degradation, as previously shown [83,84]. This proposed mechanism is schematically illustrated in Figure 11. IFITM3 has been characterised as an antiviral agent against numerous enveloped RNA viral pathogens, particularly those who enter cells through endocytosis, such as IAV and dengue (DENV). However, emerging viruses, like the Zika virus (ZIKV), still need to adapt against this immune response mechanism [85,86].

Despite the fact that LSD1 inhibition has received a lot of attention for its potential role in a variety of cancers, including prostate cancer [87], small cell lung cancer [88] and acute leukaemia [89], little is known about its potential impact on viral infections. This is the first time that the key role of LSD1 in the outcome of HCV infection is described. We suggested that HCV tightly controls LSD1 expression in order to complete its life cycle. This is because overexpression of LSD1 may play a beneficial role in the early hours of viral infection, but can also hinder infection at multiple levels later. Conversely, reduced LSD1 expression was shown to enhance HCV active replication. In this study, we proposed an intriguing antiviral mechanism, where LSD1 activates IFITM3 via demethylation, leading endocytosed HCV virions to degradation and infection to failure. Whether the HCV-mediated LSD1 oscillations over countless viral life cycles throughout chronic HCV infection elicit epigenetic changes that drive HCV-induced hepatocarcinogenesis is currently under investigation.

## 5. Conclusions

LSD1, a major epigenetic regulator of housekeeping cellular pathways, has lately emerged as an antiviral component against RNA viruses. In this study, we provide for the first time evidence of direct LSD1 involvement in the establishment of HCV infection. HCV tightly controls LSD1 expression in order to complete its life cycle. Additionally, LSD1 activates IFITM3 via demethylation, leading endocytosed HCV virions to degradation and infection to failure, rendering LSD1 a crucial component of IFN-induced immune response against HCV viral infection.

## Data Availability

Data are contained within the text and Supplementary Information.

## Supplementary Materials

The following supporting information can be downloaded at: https://www.mdpi.com/article/10.3390/cells12212568/s1, Figure S1: Constitutive LSD1 expression and activity; Figure S2: Characterisation of the stable LSD1-overexpressing cell line Hu1b; Figure S3: Ectopic expression of LSD1 inhibits HCV active replication in Huh7.5 cells; Figure S4: LSD1 overexpression reduces HCV core protein expression during infection; Figure S5: Subcellular localisation of LSD1; Figure S6: IFITM3 protein expression following interferon treatment in the presence or absence of LSD1 overexpression; Figure S7: LSD1 knockdown increases IFITM3 protein expression; Table S1: Primers used in qPCR experiments.
