# Supplementary material for: The Epigenetic Controller Lysine-Specific Demethylase 1 (LSD1) Regulates the Outcome of Hepatitis C Viral Infection
Source: Cells. 2023 Nov 3;12(21):2568. doi: 10.3390/cells12212568 (PMC10648375; doi:10.3390/cells12212568)
Supplement: Supplementary file 1 [file cells-12-02568-s001.zip › cells-2683359-supplementary.pdf]

Supplementary Material

Papadopoulou et al.

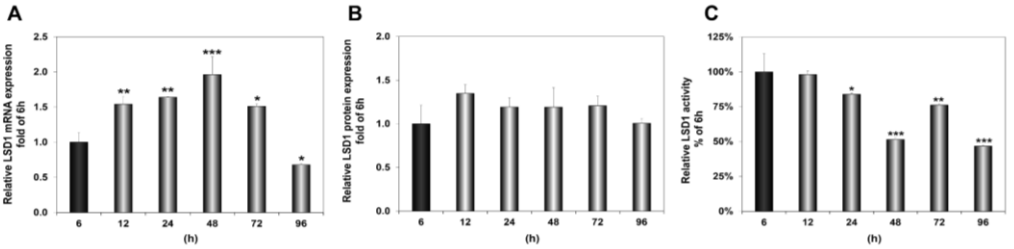

Figure S1. Constitutive LSD1 expression and activity. **(A)** Relative LSD1 mRNA expression. Huh7.5 cell cultures were used to extract total RNA that was subjected to RT-qPCR with 18S rRNA as an internal control. The relative mRNA values were plotted in expression histograms; **(B)** Relative LSD1 protein expression. Whole cell extracts were prepared from Huh7.5 cells and used in immunoblotting analysis. β-actin was used as a loading control. The blots were subjected to densitometry and plotted in expression histograms; **(C)** Relative LSD1 activity was measured in Huh7.5 whole cell extracts, normalised to total protein and plotted in histograms. The 6 h time point (black bar) was arbitrarily set as 1-fold or 100% and all other values were calculated as a ratio of this (* p-value ≤ 0.05, ** p-value ≤ 0.005, *** p-value ≤ 0.001).

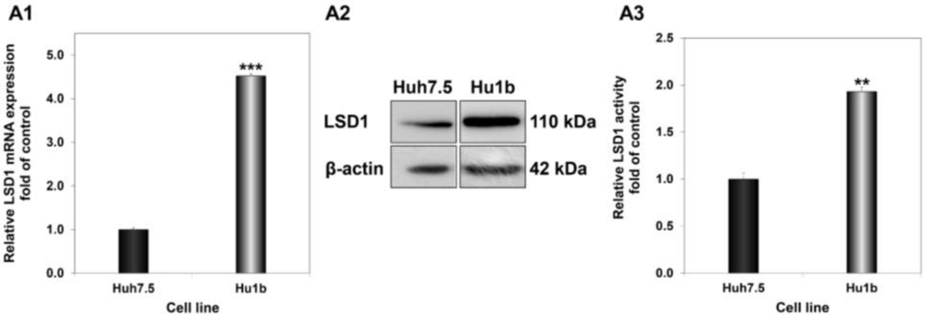

Figure S2. Characterisation of the stable LSD1 overexpressing cell line Hu1b. **(A1)** The histogram depicts relative LSD1 mRNA levels in Hu1b and control Huh7.5 cell lines. Total RNA was extracted from Huh7.5 cells and subjected to RT-qPCR. 18S rRNA was used as internal control. **(A2)** A representative Western blot of LSD1 protein expression in the Hu1b and control cell lines. **(A3)** Relative LSD1 protein in Hu1b and control Huh7.5 cell lines. Whole cell extracts were used in immunoblotting analysis and β-actin was the loading control. Blots were subjected to densitometry and plotted in expression histograms. In both graphs, values of the control cells (black bar) were arbitrarily set as 1-fold or 100% and all other values were calculated as a ratio of this (** p-value ≤ 0.005, *** p-value ≤ 0.001).

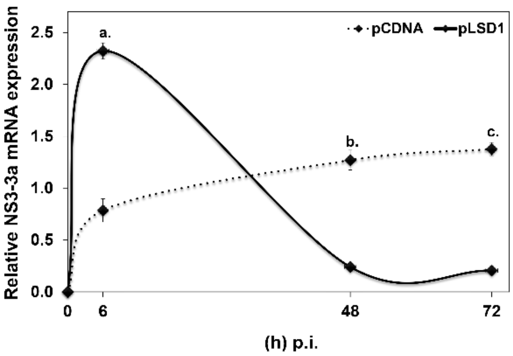

Figure S3. Ectopic expression of LSD1 inhibits HCV active replication in Huh7.5 cells. pLSD1 (bold line) and pCDNA3.1 (dashed line) plasmids were transfected into Huh7.5 cells. 30 h later, the cells were infected with HCV-3a. Cells were harvested at the selected time points and HCV NS3 mRNA expression was measured by RT-qPCR to monitor HCV active replication (stats key: a: ***, b: *** and c: ***, with *** p-value ≤ 0.001).

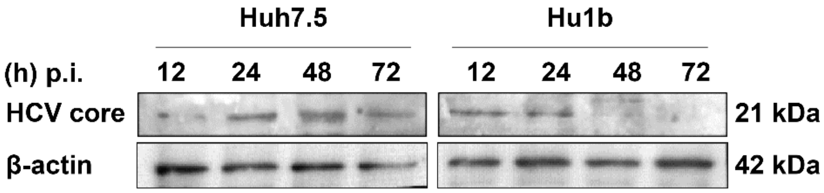

Figure S4. LSD1 overexpression reduces HCV core protein expression during infection. Whole cell extracts from infected Huh7.5 and Hu1b cells were used in immunoblotting analysis with anti-HCV core polyclonal antibody and β-actin as the loading control. This is a representative experiment of a 72 h HCV time course.

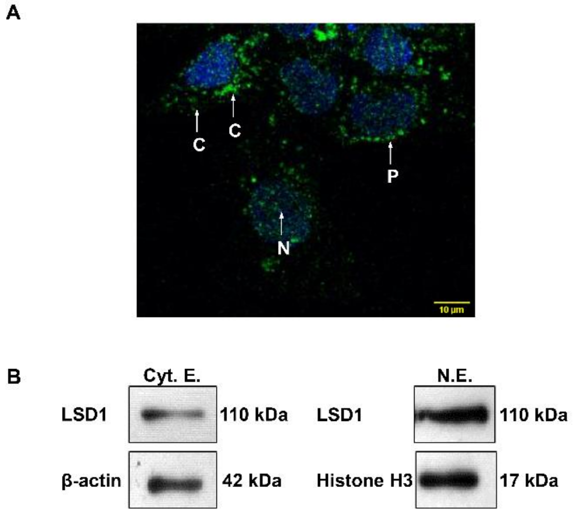

Figure S5. Subcellular localisation of LSD1. **(A)** Huh7.5 cells were subjected to immunofluorescence of LSD1, using confocal microscopy. LSD1 localisation was observed to be nuclear (N), perinuclear (P) and cytoplasmic (C). **(B)** Subcellular fractionation of Huh7.5 cells was used to produce nuclear (N.E.) and

cytoplasmic (C.E.) extracts, as indicated in the relevant methods section. The extracts were subjected to Western blotting with an anti-LSD1 monoclonal antibody to assess localisation of LSD1 in both cellular compartments. β-actin and histone H3 were used as loading controls for the cytoplasmic and nuclear extracts, respectively.

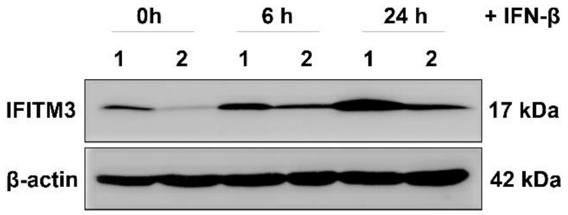

Figure S6. IFITM3 protein expression following interferon treatment in the presence or absence of LSD1 overexpression. Whole cell extracts from Huh7.5 (1) and Hu1b (2) cells before and after IFN-β treatment at the indicated time points. β-actin was used as a loading control.

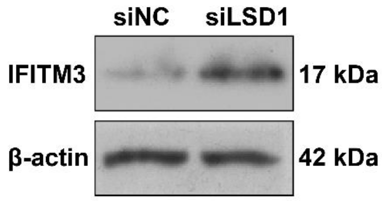

Figure S7. LSD1 knockdown increases IFITM3 protein expression. Whole cell extracts from Huh7.5 cells transfected with siLSD1 and scrambled siRNA (siNC) RNA oligonucleotides for 48 h were prepared and subjected to immunoblotting experiments to detect IFITM3 protein expression. β-actin was used as loading control.

Table S1. Primers used in qPCR experiments.

Target Gene	Forward primer	Reverse Primer
**LSD1**	5′-GCTCGGGGCTCTTATTCC-3′	5′-ATTGCAGACCAGTTTTTGGG-3′
**HCV NS3-2a**	5′-CTGCCACCCTGGGGTTTGGG-3′	5′-GCAGCCCCCATCGGCGAGAA-3
**HCV NS3-3a [43]**	5′- GCAGCGGTAAGAGCACAAAG-3′	5′-TAGGCACGCGACATGAAAGA-3′
**18S rRNA [S1]**	5′-CTCAACACGGGAAACCTCAC-3′	5′-CGCTCCACCAACTAAGAACG-3′

Supplementary References

43 V. Valiakou *et al.*, "Differential Expression of the Host Lipid Regulators ANGPTL-3 and ANGPTL-4 in HCV Infection and Treatment," *Int. J. Mol. Sci.*, vol. 22, no. 15, p. 7961, Jul. 2021, doi: 10.3390/ijms22157961.

S1 E. Tsitoura, J. Thomas, D. Cuchet, K. Thoinet, P. Mavromara, and A. L. Epstein, "Infection with herpes simplex type 1-based amplicon vectors results in an IRF3/7-dependent, TLR-independent activation of the innate antiviral response in primary human fibroblasts," *J. Gen. Virol.*, vol. 90, no. Pt 9, p. 2209, Sep. 2009, doi: 10.1099/vir.0.012203-0.
